# An evaluation of ascitic calprotectin for diagnosis of ascitic fluid infection in children with cirrhosis

**DOI:** 10.1186/s12887-022-03433-9

**Published:** 2022-06-30

**Authors:** Naser Honar, Najmeh Nezamabadipour, Seyed Mohsen Dehghani, Mahmood Haghighat, Mohammad Hadi Imanieh, Maryam Ataollahi, Nader Shakibazad, Hazhir Javaherizadeh

**Affiliations:** 1 Neonatal Research Center, Shiraz University of Medical Sciences, Shiraz, Iran; 2 Gastroenterohepatology Research Center, Shiraz University of Medical Sciences, Shiraz, Iran; 3 Department of Pediatrics, Bushehr University of Medical Sciences, Bushehr, Iran; 4 Alimentary Tract Research Center, Clinical Sciences Research Institute, Ahvaz Jundishapur University of Medical Sciences, Ahvaz, Iran

**Keywords:** Ascites, Calprotectin, Liver

## Abstract

**Background:**

The most common infection in children with the hepatic disease with or without cirrhotic ascites is spontaneous bacterial peritonitis (SBP), which occurs in the absence of an evident intra-abdominal source of infection. The present study aims to assess the value of calprotectin in ascitic fluid in the diagnosis of ascitic fluid infection in children with liver cirrhosis.

**Materials and methods:**

In this cross-section study, 80 children with underlying liver disease who attended the Hepatology and Emergency Department in Shiraz University Hospitals were studied. All the patients were evaluated by a thorough history, clinical examination, laboratory investigations, diagnostic paracentesis with PMNLs count, and Calprotectin, which was measured in 1 mL ascitic fluid by ELISA.

**Results:**

Thirty-five patients (43.75%) were diagnosed with ascitic fluid infection. Of these children 6 cases had positive ascitic fluid culture (SBP). Calprotectin was high in AFI patients with a statistically significant difference in AFI patients compared to non-AFI patients. The cut-off levels were 91.55 mg /L and the area under the curve was 0.971. So it can serve as a sensitive and specific diagnostic test for detection of AFI in children with underlying liver disease.

**Conclusion:**

Elevated ascitic calprotectin levels in cirrhotic patients are a diagnostic and reliable marker for the detection of AFI and are considered a surrogate marker for PMN.

## Introduction

Ascites is a major complication of cirrhosis, indicating the progression of cirrhosis and poor prognosis. The mortality rate of patients with ascites due to the cirrhosis is up to 50% over 2 years [1]. The Spontaneous Bacterial Peritonitis (SBP) is known as an acute bacterial infection of ascites, so that no source of intra-abdominal infection can be diagnosed [2].

The calprotectin as a biomarker with non-invasive measurement has been used to identify gastrointestinal inflammation. Calprotectin is a calcium- and zinc-binding protein with antimicrobial activity that is almost exclusively detected in neutrophils. Its presence in body fluids is proportional to the influx of neutrophils. Calprotectin levels can be obtained by the ELISA test. Burri et al. reported that the calprotectin measurement in ascites is well associated with the PMN count and reliably indicates a level of ≥ 250 cells/ mm in ascites, as a standard marker for detection of SBP [3, 4]. Studies have been conducted on the use of ascitic calprotectin for the diagnosis of SBP in patients with cirrhosis. Recent studies (2015- 2016) have found that rate of ascitic calprotectin was significantly higher in patients with cirrhosis with SBP than people without SBP [5]. There is limited research about role of calprotectin in children with ascites [6] and most of published researches were conducted in adults patients. So, the present study aimed to evaluate ascitic calprotectin value in the diagnosis of ascitic fluid infection in children with cirrhosis.

### Research method

The present study was cross-sectional and included all patients with ascites which was paracentesized in the pediatric gastrointestinal and emergency department of Namazee Hospital of Shiraz during January 2016 to January 2017. Children with liver cirrhosis and ascites were included in this study. The decision to perform the paracentesis was made on the basis of clinical findings by the relevant physician. Exclusion criteria included the age of over 18, abdominal surgery in less than 3 months, and ascites without liver disorders. Patients who receive antibiotocs were excluded. Standard disease history, clinical symptoms, and demographic data were collected from all patients. The present study was based on approved principles by the Ethics Committee of Shiraz University of Medical Sciences. Sampling was performed only in patients with paracentesis indications.

The final diagnosis of liver disorder was done by the specialized pediatric gastroenterology team based on the obtained information from patients’ history during the referral, the patients’ clinical symptoms, results of all paraclinical examinations, and patients’ responses to treatment.

### Ascitic fluid paracentesis

The paracentesis was performed under aseptic conditions in Supine position and preferably from the midline, below the navel or McBurney’s point on the left or right for patients. In the case of ascitic fluid deficiency and on the basis of physician’s diagnosis, the primary ultrasound was performed to evaluate the appropriate point of paracentesis. In some patients, the paracentesis was performed by a radiologist under the guidance of an ultrasound probe.

Ascitic fluid paracentesis was done in strile setting. All specimens were collected for patient’s clinical examination; and the following basic laboratory assessments were done: white blood cell count; Polymorphonuclear (PMN) cell count; Glucose levels; protein; Lactate dehydrogenase (LDH) albumin, and ascitic fluid culture for patients. The samples were kept at -4 °C until laboratory kit preparation in order to examine the calprotectin level of ascitic fluid.

According to the results of PMN count and ascitic fluid culture, Culture negative neutrocytic ascites (CNNA) was defined as PMN > 250 with negative ascitic fluid culture. Sponatneous bacterial peritonitis was defined as PMN > 250 with positive ascitic fluid culture. Both of them was considered as ascitic fluid infection (AFI) [7].

### Laboratory measurements

All laboratory studies were conducted by senior laboratory staff, who were unaware of patients’ history and calprotectin levels, in the central laboratory of Namazi Hospital of Shiraz. The total leukocyte cell count and its types in ascitic fluid were determined by a manual method.

### Cell count

First, the ascitic fluid was diluted if necessary and was well mixed to spread cells uniformly in the liquid. A lamellar was put on a neobar lam; and 10 of ascitic fluid was put under lameller by a sampler to totally cover lam and not get out of it. The neobar lam was put motionless under the microscope for 2 min, so that the cells were distributed evenly. The standard Neobar calculation formula, which was used to count blood cells, was also used to determine amount of cells per microliter of ascitic fluid. The cytopathological analysis was performed at least once for all study participants who experienced recurrent paracentesis.

### Calprotectin measurement

Ascitic calprotectin was measured using the ELISA method according to the kit manufacturer’s instructions. The kit was based on the sandwich enzyme-linked immune-sorbent assay technology. The anti-calprotectin antibody covered 96-well plates, and the Conjugated Anti-Calprotectin antibody to biotin was used as the detected antibody. Standards, test specimens, and conjugated detected antibodies to the biotin were added later to wells and rinsed with buffer. Horseradish Peroxidase (HRP)-Streptavidin was added and unbound conjugates were rinsed with buffer. Tetramethyl benzidine (TMB) was used to demonstrate the HRP enzyme reaction. TMB was catalyzed by HRP and produced a blue product that turns yellow after adding an acidic stop solution. Yellow density was proportional to the amount of obtained Calprotectin of sample on the plate. In summary, 10 µl of ascitic fluid samples were diluted as 1: 50 ratio in incubation buffers, and 100 µl were applied to a microtitre plate coated with a highly specific monoclonal capture antibody for calprotectin polymeric and heterodimeric complexes. The TMB Chromogenic substrate was added after incubation, rinsing, and re-incubation with the diagnostic antibody that was conjugated to HRP Peroxidase. The reaction was terminated by a stop solution, and optical absorption (optical density at 450 nm) was measured by spectrophotometry.

### Statistical analysis

The obtained data from the present study were analyzed using SPSS21 and results were presented as mean ± standard deviation for the quantitative variable and as a percentage for qualitative variables. Statistical tests, Spearman’s for non parametric variable, Chi-square and T-test, were used if necessary and the *P*-value was less than 0.05 as a significant relationship among the studied variables.

## Results

Eighty patients were studied in the present study. Forty-six patients were female and 34 were male. Ascitic fluid infection (SBP and CNNA) was diagnosed in 35(43.75%) of patients. Ascitic fluid culture was positive in 6 cases. The Ascitic calprotectin level was measured in patients with and without AFI and compared in two groups. Furthermore, the level of Calprotectin was compared with laboratory criteria of ascitic fluid as well as in various underlying liver diseases. Furthermore, laboratory findings and clinical findings of patients were measured and compared during admission in two groups with and without AFI. Calprotectin levels were compared with clinical and laboratory findings of patients and statistical relationships between the variables were determined. Etiology of liver disease was seen in the Table 1.

**Table 1:** Etiology of liver cirrhosis

Etiology	N
Biliary atresia	23
Wilson’s disease	9
Autoimmune hepatitis	8
PFIC	7
Budd-Chiari syndrome	6
Tyrosinemia	6
Neonatal hemochromatosis	3
Galactosemia	3
GSD type IV	﻿3
Alagile syndrome	1
Other	11

Abdominal pain was present in 31.4% of patient with AFI and 33.3% of children without AFI( *P* = 0.85). Vomiting was present in 37.1% of children with AFI and 15.6% without AFI( *p* = 0.027). Fever was detected in 48.6% of children with AFI and 31.1% without AFI(*p* = 0.11). Poorfeeding was seen in 51.4% of children with AFI and 37.8% without AFI(*p* = 0.22). Lethargy was found in 48.6% of children with AFI and 48.9% without AFI(*p* = 0.97). Decreased level of consciousness was seen in 48.6% of cases with AFI and 28.9% of cases without AFI (*p* = 0.07).

There is a significant positive correlation between calprotectin and variables, namely WBC (*p*-value < 0.001, *r* = 0.76), PMN (*p*-value < 0.001, *r* = 0.78) and LDH (*p*-value = 0.002, *r* = 0.33). In other words, the increased WBC, PMN and LDH raised the calprotectin variable(Table 2).

**Table 2:** Mean level and laboratory factors of ascitic fluid and their correlation with Calprotectin level

	**Mean level of calprotectin(μg/dL)**	**Standard Deviation**	**Correlation with Calprotectin level (*P*-value)**
Calprotectin	437.64	± 667.18	
WBC	680.67	± 1074.84	**0.000**
PMN	570.1125	± 1010.37	**0.000**
Glucose	100.52	± 29.09	**0.934**
Protein	1.03	± 0.65	**0.024**
Albumin	0.67	± 0.49	**0.272**
LDH	208.55	± 174.41	**0.002**

**Fig. 1:**
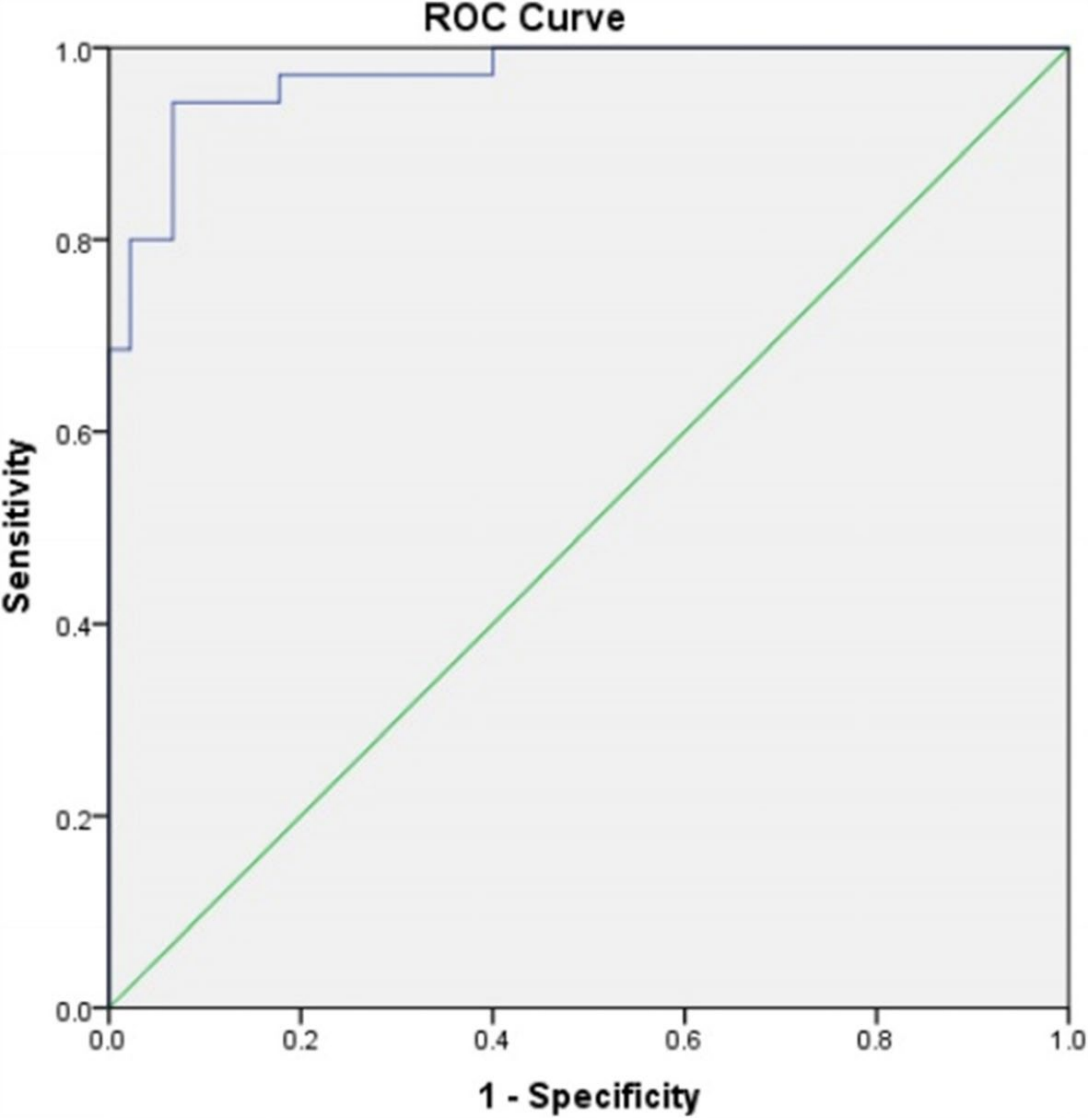
The area under the curve in the ROC curve in the evaluation of sensitivity and specificity of calprotectin variable in the SPB diagnosis

The ROC curve indicates the sensitivity and specificity of a model or criterion in measuring a variable (Fig. 1). The area under the curve close to 1 indicates the high sensitivity and specificity of a criterion in measuring a variable. According to the area under the graph in the ROC curve in the present study (numerical value of 0.971) in the evaluation of calprotectin criteria in the assessment of presence and absence of SBP, the criterion has high sensitivity and specificity in the diagnosis of SBP (Table 3).

**Table 3:** Cut off point range of calprotectin level, indicating the sensitivity and specificity of greater than 90%

Calprotectin Level mg/L	Sensitivity	Specificity
83.95	94.3	88.9
87.50	94.3	91.1
91.55	94.3	93.3
107.25	91.4	93.3
122.50	88.6	93.3

According to the results of Table 3, the value of the Cut-off point is equal to 91.55 with the highest sensitivity and specificity for calprotectin in diagnosing the existence of SBP. The above table indicates that the calprotectin range is 83.95 to 122.50 and has the greatest sensitivity and specificity in the diagnosis of SBP.

Our results showed that only 2 out of 35 patients with AFI had calprotectin levels lower than the Cut-off point in the study. Furthermore, only 3 out of 45 patients with negative SBP had calprotectin levels more than the Cut-off point.

**Fig. 2:**
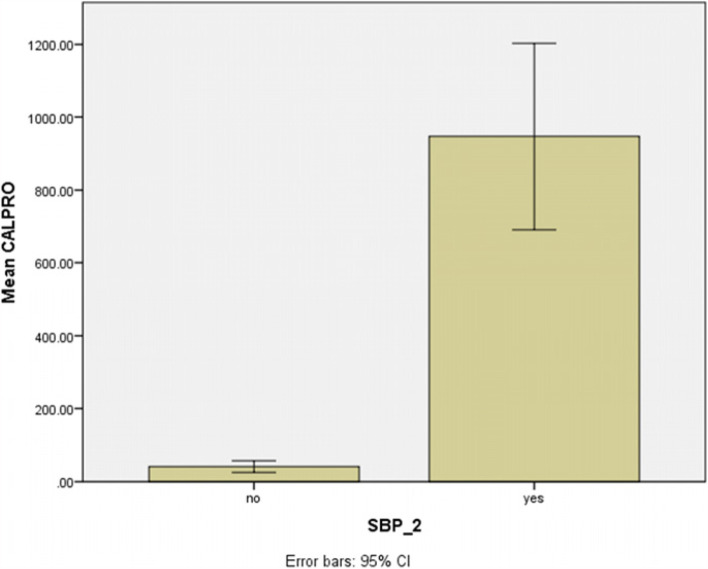
The Confidence Level Diagram in the evaluation of mean calprotectin levels in patients with and without AFI

The above figure shows the mean value of calprotectin as well as its concentration distribution in 95% of people with positive and negative SBP (Fig. 2).

## Discussion and conclusion

Abdominal ascites are often seen in patients with liver disorders and with and without cirrhosis and may cause transmission of bacteria and increased risk of SBP. SBP is rare in outpatients, but when it occurs, it often requires hospitalization to treat the disease.

In the present study, 35 out of 80 patients (43.7%) had SBP. In the study by Singh et al. [8], AFI was detected in 31% of children which is lower than our study. In the study by Srivastava et al., 28.6% of children had SBP/CNNA in their study [7]. The rate of AFI in our study was higher than their study, and this may be due to selection bias, or different severity of liver disease among these studies.

However, the c-reactive protein (CRP) and WBC level measurement can be detailed tests in the diagnosis of SBP due to a persistent systemic inflammatory response, and the use of moderate platelet volume, but the diagnosis of SBP is still based on the paracentesis. In general, symptoms of the SBP are non-specific, and current guidelines recommend that paracentesis should be performed in all patients with ascites or suspected of having SBP to prevent abdominal infection. Diagnosis of SBP in patients with cirrhosis is based on the number of PMN > 250/μL in the paracentesis fluid with or without bacterial positive culture. The criterion is known as the most sensitive criterion to the diagnosis of SBP. Its diagnosis solely based on the result of bacterial culture is unreliable because up to 60% of patients are reported as a negative culture as the number of PMN raised. In the present study, the bacterial culture was positive for only 6 patients out of 35 ones with AFI (84.86% of patients with AFI showed negative culture results: CNNA). According to research by Burri et al. [3], the positive culture result of paracentesis fluid was related to an increased number of PMN. In the present study, calprotectin level was measured in paracentesis fluid from 80 consecutive patients. Ascitic Calprotectin levels were well and significantly correlated with PMN counts, and samples with PMN > 250/μL showed higher ascitic calprotectin levels than samples with PMN < 250/μL. Ascitic calprotectin reliably predicts PMN counts [3].

According to the ascitic fluid analysis, 35 patients had AFI; 49.2% of patients with AFI were male and 57.1% were female. With a mean age of 44.81 months, there was no significant difference between cases with and without AFI according to the mean age and sex. Makhlouf et al. studied the ascitic calprotectin and found no significant correlation between the prevalence of sex and age in patients with a prevalence of SBP [9]. The most common clinical symptoms in the SBP group were Poor feeding (51.4%), Fever, lethargy, and decreased level of consciousness (48.6%). The presence of none of the clinical symptoms did not show any significant correlation with the presence and absence of SBP. In a study by Makhlouf et al., abdominal pain and then fever, hepatic encephalopathy, and abdominal tenderness were the most common clinical manifestations of the SBP [9].

Calprotectin is a protein, derived from the decomposition of neutrophils, and can be found in the abdominal fluid and stool and increases in infectious and inflammatory conditions. Previous studies found that the ascitic calprotectin was useful in the diagnosis of SBP in patients with liver impairment [10]. According to the assessment of sensitivity and specificity of this test in the diagnosis of SBP and based on the ROC curve, the accuracy of this test was very high (area under the curve of 0.971). The results were consistent with research by Burri et al. [3]. In the study, the measurement of ascitic calprotectin was performed by ELISA and POC methods; and the ROC curve was used to examine the accuracy of both methods in the diagnosis of SBP by measuring the Calprotectin concentration. Like the present study, the area under the ROC curve was 0.977 in a study by Burri [3].

Results of the present study indicated that an increase in calprotectin level raised the specificity of the test while decreasing its sensitivity. The Calprotectin range of 83.95 to 122.5 µg/dL indicated the sensitivity and specificity of higher than 88% in the diagnosis of SBP. The Cutoff point of 91.55 led to the maximum sensitivity of 94.3% and specificity of 93.3%. The value was 63 µg/ dL in a study by Burri, and 44.5 µg/ dL in a study by Abdel- Razik et al. [11]. In the evaluation of calprotectin level in ascitic fluid in patients with liver failure in Egypt, Heikl et al. considered a concentration of 78.3 Μg/dL as the Calprotectin Cutoff in the SBP diagnosis [12].

Results of the present study confirmed findings of research by Parsi et al. [13]. Our results indicated that the measurement of calprotectin, a leukocyte-specific protein, might serve as an alternative marker for the PMN count in the ascitic fluid.

In the present study, the calprotectin level of Ascitic fluid showed a significant correlation with the PMN count (*P* < 0.001), and thus the ascitic calprotectin was a reliable alternative to the PMN count. Ali et al. found a significant negative correlation between calprotectin concentration and blood albumin level. In the present study, calprotectin levels showed a significant correlation with markers such as LDH, WBC, and ascitic fluid protein that increased in infection of the fluid. Calprotectin levels increased significantly in patients with positive SBP compared to those without the SBP (*P* < 0.001). Fernandes et al. [14], Elbanna A. et al. [15], and Ghweil et al. [10] found similar results. According to the study by Bender et al., measurement of calprotectin level in ascetic fluid should be done for SBP [16]. In the study by Josifovikj et al., ascites calprotectin level is a good predictor for diagnosis and follow up of patients with SBP [17].

In the evaluation of calprotectin levels in underlying diseases of liver failure, the highest mean concentration of calprotectin was observed in patients with GSD-IV, and the lowest concentration was seen in neonatal hepatitis patients. Results of the present study indicated that calprotectin concentration was not significantly correlated with an underlying factor of hepatic cirrhosis. In the evaluation of the correlation of calprotectin concentration with the result of ascitic fluid culture, results of the present study indicated that concentrations of calprotectin were higher in patients with positive ascitic fluid than those with negative culture; however, the difference was not statistically significant. Furthermore, there was no statistically significant correlation between patients’ clinical symptoms and ascitic calprotectin level.

A few studies have evaluated the concentration of abdominal fluid calprotectin in children with liver impairment to investigate its correlation with the presence and absence of SBP. Results of the present study indicated that the laboratory finding could be used as an accurate marker in the examination of the presence of SBP. The area under the ROC curve indicated high accuracy of the test in the diagnosis of counting PMN ≥ 250. Furthermore, the cutoff point Eof equal to 91.55 µg/dL led to the sensitivity and specificity of greater than 90% in the diagnosis of patients with positive SBP. According to the results, Calprotectin could be used as a marker in the diagnosis of SBP in addition to PMN counting. Both variables also showed significant correlations with other laboratory markers, which increased in the presence of SBP, such as WBC, LDH, and ascitic fluid protein. None of the clinical findings and underlying causes of diseases were correlated with the prevalence of SBP and calprotectin concentrations.

### Limitation

Single-center study.

## Data Availability

The datasets used and/or analysed during the current study available from the corresponding author on reasonable request.

## Abbreviations

AFI: Ascitic fluid infection
CNNA: Culture Negative Neutrocytic Ascites
GSD: Glycogen storage disease
SBP: Spontaneous bacterial peritonitis
WBC: White blood cell
